# A psychosocial intervention to reduce gender-based violence and antepartum depressive symptoms in pregnant women in Kisumu County, Kenya: a quasi-experimental study

**DOI:** 10.11604/pamj.2018.29.11.13596

**Published:** 2018-01-04

**Authors:** Redempta Kalekye Mutisya, Kenneth Ngure, Christina Mwachari

**Affiliations:** 1 School of Public Health, Jomo Kenyatta University of Agriculture and Technology, Nairobi, Kenya; 2 Department of Population and Reproductive Health, School of Public Health, Kenyatta University, Nairobi, Kenya; 3 Kenya Medical Research Institute, Nairobi, Kenya

**Keywords:** Gender-based violence, antepartum depression, pregnant women, intervention

## Abstract

**Introduction:**

Gender-based violence during pregnancy and its associated adverse health effects are disproportionately higher in developing countries like Kenya where screening for and management of gender-based violence is currently not part of routine antenatal care. This study assessed the effect of a psychosocial intervention on gender-based violence and antepartum depressive symptoms in pregnant women.

**Methods:**

This quasi-experimental study compared gender-based violence and antepartum depression scores of 288 pregnant women in the two arms; one exposed to a psychosocial intervention and another receiving usual antenatal care. We used analysis of covariance to estimate the intervention effect and Chi-square to test the equality of proportions.

**Results:**

The difference between the psychosocial intervention and the usual antenatal care group in the total intimate partner violence and physical violence scores was a significant, with small effect sizes of partial eta = 0.196 and 0.305 respectively. The two arms did not differ in terms of the proportion of women reporting other acts of gender-based violence by intimate and non-intimate partners post-intervention. The intervention group had significantly lower mean depression scores compared to the usual care group, post-intervention, with a medium effect size of 0.500.

**Conclusion:**

This intervention aimed at reduction of gender-based violence and improvement of mental health of pregnant women is promising. Primary health care facilities in resource-constrained settings can take advantage of local capacity to deliver focused non-specialized psychosocial support to pregnant women experiencing violence.

## Introduction

Gender-based violence (GBV) in this study referred to a self-reported experience of one or more acts of physical, sexual, psychological and economic violence by intimate partners, family members or any other persons (non-partner violence) in the 12 months preceding the pregnancy and during the current pregnancy. Such violence is pervasive and a systemic public health problem affecting women of all socio-economic and cultural groups throughout the world at a high cost to the individual and society [1]. Kenya has among the highest prevalence of GBV in Sub Saharan Africa with 47% of ever-married women reporting a lifetime prevalence of physical or sexual abuse from a spouse [2]. Kisumu County was part of the former Nyanza Province that had the highest prevalence of physical violence (57.1%) and sexual violence (22%) among women aged 15-49 years in the country [2]. GBV during pregnancy is also prevalent in many parts of the world [2-4]. A multi-country survey reported the prevalence of intimate partner violence (IPV) during pregnancy to range between 4-32%, with low-income countries reporting a higher prevalence of 14-32% [5]. A rural clinic in Kisumu County found 37% of pregnant women had been physically, sexually or psychologically violated [3]. These statistics are evidence that violence during pregnancy is more prevalent than most other conditions that women get screened for during pregnancy [5]. Perinatal depression occurs during pregnancy or postnatal with serious implications on both maternal and infant outcomes [6, 7]. Maternal depression is associated with preterm birth, low birth weight and pregnancy complications [8-10] as well as inadequate weight gain [11]. Depression during pregnancy has also been associated with gestational hypertension and subsequent preeclampsia [12, 13], spontaneous abortion [14] and bleeding during gestation [15].

Greater emphasis has however been placed on perinatal depression during the postpartum period and relatively less attention has been paid to depression in the antepartum period [16] in spite of the substantial prevalence (19-25%) of the same in low-and-middle-income countries [17]. Experience of GBV during pregnancy has been cited as an important risk factor for antepartum depressive symptoms [18-21]. Other adverse health outcomes linked to violence during pregnancy include miscarriage [22], premature labor or delivery [23], low birth weight [24, 25], higher levels of depression after pregnancy [26, 27], anxiety, low self-esteem/confidence [28], human immunodeficiency virus (HIV)/ acquired immunodeficiency syndrome (AIDS), sexually transmitted infections, injury [29] and maternal deaths [30, 31]. Most developed countries have mechanisms that take advantage of the pregnancy window to identify and support women experiencing violence and even though similar mechanisms have the potential to work in Kenya [3], they are currently non-existent. Emphasis has been made on the need to evaluate the effectiveness of facility-based interventions in reducing and mitigating the adverse effects of violence during pregnancy in resource-poor settings [3, 5]. However, to date, very few such studies have been done [32], particularly in primary facilities which are utilized by a majority of the pregnant women. The use of trained non-professionals to deliver focused non-specialized support for GBV may be a viable option [33-35] in resource-constrained settings as is the case in primary health care facilities in Kenya. The effectiveness of this approach has however not been assessed. This study took advantage of the antenatal care (ANC) window to identify violated pregnant women for follow-up support because 95% women in Kenya come into contact with health care providers during pregnancy [2]. The purpose of this study was to assess the effect of a psychosocial intervention on GBV and antepartum depression in pregnant women in Kisumu County.

## Methods

**Study design**: This study was a quasi-experiment carried out in 12 public primary health care facilities in Kisumu County. One facility in each of the six sub-counties was randomly assigned to have the intervention group and the other the usual antenatal care group.

**Subjects**: The study population comprised of pregnant women aged 18-45 years seeking ANC services in the selected health facilities. Other eligibility criteria were; a positive GBV score, being in the first or second trimester of pregnancy, no intent to leave the area or change the ANC facility during the study period and mental competence to provide consent. We excluded pregnant women accompanied to ANC by a spouse/partner or other persons and women who requested referrals for specialized treatment.

**Sample size determination**: WE used the Power Sample size (PS) calculator version 3.1.2 to determine the sample size [36]. The study aimed to detect a difference of 16% in GBV or depression at the end of the intervention (an improvement for 1 in 6 women was considered clinically important) [33]. The statistical power was 80% at 95% level of confidence. The prevalence of GBV in pregnant women was 37% in the control group from a previous study [3]. With one woman in the control group per intervention subject (m = 1), the sample size was 137. After factoring attrition rate of 5%, the required sample size increased to 144.

**Measures**: Interviews took place in a quiet private room within/close to the ANC clinic in English or the local language (Dholuo) depending on the client's preference. Screening for GBV was done using the Abuse Assessment Screen (ASS) [37]. All pregnant women attending ANC in the study sites responded to questions on whether they had ever been slapped, kicked, forced into sexual activities or emotionally abused by their intimate partner or anyone at any time, during the past year and during the current pregnancy. A “yes” to any of the five questions in the ASS was considered as exposure to GBV while “no” answers to all questions indicated an absence of GBV. Reliability of the ASS was measured using Cronbach's alpha (α = 0.896). We obtained baseline and post-intervention data from eligible participants in both arms. Assessment for GBV was by use of a modified pregnancy version of the Composite Abuse Scale, (CAS) [38]. Cronbach's alpha for CAS were; total CAS α = 0.917, severe combined violence α = 0.701, physical violence α = 0.889, emotional violence α = 0.831 and harassment (four items) α = 0.655.

The Edinburgh Postnatal Depression Scale (EPDS) [39] was used to measure antepartum depression, α = 0.726. To assess violence in the post-intervention interview, research assistants rephrased the questions to allow participants respond for the period after the baseline assessment and the time of the last interview. For the CAS, the research assistant asked: “Since the time we met for the first interview has your husband/partner/anybody else.”The risk of contamination between groups was minimal because the participating health centers were far apart. A health center in Kenya provides primary health care services and serves a given catchment area hence a low likelihood that a participant would leave the facility near her home for one that is further away. Participants' responses in the exit interview corroborated our view. The research assistants in the intervention facilities worked closely with the ANC nurse to minimise the waiting time at the clinic and escorted out each participant after a psychosocial session/interview to reduce potential interaction between participants. The assistants were not aware of the study hypotheses since they were not involved in its design.

### Description of the care

**Psychosocial intervention group**: Participants in the intervention arm received the usual ANC services and psychosocial support as part of follow-up. Trained research assistants with previous experience in social work both in the community and with patients in a healthcare setting delivered the intervention. Research assistants made the participants comfortable and relaxed in a quiet private room and proceeded to create rapport. The intervention adopted a secondary prevention approach for early detection and reduction or elimination of GBV during pregnancy. It has its theoretical basis in Dutton's empowerment model [40] which is premised on the assumption that perpetration of GBV particularly in intimate relationships is part of a pattern of coercive control [41]. Dutton emphasized the need to increase abused woman's independence and sense of control over life through increasing safety and enhancing decision-making and problem-solving ability. Parker and colleagues opined that women have a superior understanding of their context and know what is best for them. Abused women thus need as part of help, an opportunity to express their feelings to empathetic persons in a non-judgemental environment, while being allowed autonomy to make their own decisions [42].

The psychosocial intervention entailed providing of unbiased information on the meaning and magnitude of GBV, the GBV cycle and the adverse effects of violence during pregnancy. Research assistants actively interviewed, listened empathetically, validated feelings and encouraged the participants. Research assistants also pointed out that the woman was not to blame for the violence meted on her. The intervention also included a safety assessment and facilitation to identify measures that participants could take to enhance their safety based on a safety protocol by McFarlane and Parker [43]. Participants received resource cards listing local persons/organizations from where they could seek further assistance for GBV in addition to information on the availability of coordinated referrals for specialized counselling services. The research assistants aimed to hold at least three 30-35 minutes psychosocial support sessions with each participant before conducting the last intervention interview. The first session took place within 2-3 weeks after the baseline interview and the remaining sessions spread over a 4-5 month period. The sessions coincided with ANC appointment dates whenever possible and special appointments negotiated with participants where the former was not possible and in case of missed ANC visits. The cost of transport was reimbursed and participants were given a snack whenever they availed themselves for a special appointment. The provisions, which were modest and not the norm were unlikely to be a source of undue influence on the participants.

**Usual ANC group**: Participants in the comparison group received the usual ANC services and a card listing local persons/organizations where help for GBV could be sought. These women held one psychosocial session with the research assistant after the final interview.

**Data management and analysis**: All study participants received unique identification numbers recorded on the questionnaire. Data were analysed using the Statistical Package for Social Scientists (SPSS) version 20.0 (Armonk, New York). One-way between-groups analysis of covariance was used to estimate the intervention effect on the quantitative outcomes with baseline scores as the covariates and the Chi-square used to test for equality of proportions for categorical outcomes. The independent variable was the type of care and the dependent variables were the total CAS scores, subscale scores and EPDS scores. Other dependent variables were economic violence, physical and sexual violence by non-intimate partners and perpetration of violence against an intimate partner. Preliminary checks conducted to ensure that the assumptions of reliable measurement of the covariate, normality and linearity, homogeneity of variances and homogeneity of regression slopes showed no violations. Baseline data were used in case a participant lacked post-intervention data. Research assistants emphasized that provision of ANC and any other services were not dependent upon participation in the study. Deliberate measures taken to protect participants and the research team from unintended harm included an emphasis on the need to keep one's participation in the study secret, issuing resource cards alongside other ANC pamphlets from the ministry of health, recruiting and interviewing only participants unaccompanied to ANC by a spouse/partner or any other person. All participants gave a written informed consent to participate in this study. The Scientific and Ethics Review Unit of the Kenya Medical Research Institute and the Board of Post Graduate Studies, Jomo Kenyatta University of Agriculture and Technology approved the study.

## Results

**Intervention fidelity**: A total of 288 participants were recruited, 144 in each arm. Two participants in the usual care arm did not complete the baseline assessment (one requested for referral for specialized counseling services and one did not return for the baseline and could not be traced throughout the study period). All 142 completed the last interview. In the intervention arm, three (3) women requested for referral and two (2) others were lost to follow-up after completing the baseline. All except one completed the post-intervention interview. More than three-quarters of the participants, 109 (77.3%) completed all the three supportive sessions, 22(15.6%) completed two supportive sessions and 10(7.1%) completed one session before taking the last interview.

**Selected baseline characteristics by type of care**: A comparison between the usual care and the intervention group on some baseline characteristics and exposure to violence between parents/guardians found no significant differences (Table 1).

**Table 1:** Selected baseline characteristics of participants by type of care

Characteristic	Usual Care [n=142 ]	Intervention Group [n=141]
	n (%)	n (%)
Age at Sexual Debut (Years; Mean±SD)	16.2±2.06	16±2.51
**Age**		
≤ 22 years	47(33.1)	60(42.6)
23-26 years	50(35.2)	37(26.2)
+27 years	45(31.7)	44(31.2)
**Currently Living with a Man/Partner**		
No	21(14.8)	18(12.8)
Yes	121(85.2)	123(87.2)
**Age Difference with Spouse/Partner**		
0-4 years	77(54.2)	63(44.7)
> 4 years	65(45.8)	78(55.3)
**Respondent’s Level of Education**		
Primary or Less	103(72.5)	93(66.0)
Post- Primary	39(27.5)	48(34.0)
**Partner’s Level of Education**		
Primary or Less	89(62.7)	83(58.9)
Post-Primary	53(37.3)	58(41.1)
**Woman has Own Income Source**		
No	78(54.9)	82(58.2)
Yes	64(45.1)	59(41.8)
**Number of Children**		
< 3	121(85.2)	113(80.1)
³ 3	21(14.8)	28(19.9)
**Respondent Drinks Alcohol**		
No	117(82.4)	113(80.1)
Yes	25(17.6)	28(19.9)
**Presence of Child not Born to Current IP**		
No	92(64.8)	87(61.7)
Yes	50(35.2)	54(38.3)
**Witnessed Violence as child**		
No	54(38.0)	51(36.2)
Yes	88(62.0)	90 (63.8)

**GBV at baseline**: IPV and violence by a non-intimate partner (non-IP) are reported for the past 12 months referred hereafter as “recent” and during the current pregnancy hereafter “current”. The usual care group had higher and more varied mean scores for recent total IPV and IPV in all four subscales. The groups differed significantly in the all recent IPV scores except in severe combined violence. For current IPV, the groups had significantly different scores except in physical violence (Table 2). A general trend of the decrease in scores between the baseline and the end of the study was observed in both groups with the largest decline in scores (8.5 points) occurring in the intervention group's total IPV score. The intervention group reported significantly lower recent economic violence (being chased away from home by an intimate partner (IP) compared to the usual care group, p = 0.020, but had significantly higher prevalence of physical violence by a non-IP, (p = 0.013). The groups did not differ in terms of respondents' recent perpetration of violence against an IP. The proportion of women reporting current acts of violence such as partner's refusal to use a condom (p = 0.003) and physical violence by a non-IP (p = 0.014) was significantly higher in the intervention group compared to the usual care group. The groups, however, did not differ in other forms of current abuse by intimate and non-IPs (Table 3).

**Table 2:** IPV At baseline by type of care

IPV Measure	Recent IPV				Current IPV			
	Usual Care, n=142 Mean[SD]	Intervention Group, n=141 Mean[SD]	Mean difference	t (p-value)	Usual Care, n=142 Mean [SD]	Intervention Group, n=141 Mean[SD]	Mean difference	t (p-value)
Total CAS IPV Score	33.89 (21.9)	28.95 (12.88)	4.94	2.521 (0.012)	35.18 (22.8)	26.39 (12.8)	8.79	4.23 (< 0.001)
Severe Combined Violence	5.61 (4.51)	5.19 (3.21)	0.42	0.979 (0.328)	5.62 (4.8)	4.24 (3.1)	1.38	3.070 (0.002)
Physical Violence	8.12 (6.49)	11.63 (3.11)	-3.51	-6.325 (< 0.001)	8.17 (7.2)	10.36 (3.4)	-2.19	-3.585 (< 0.001)
Emotional Violence	14.33 (9.53)	10.66 (6.68)	3.67	4.088 (< 0.001)	15.69 (10.6)	10.76 (6.5)	4.93	5.232 (< 0.001)
Harassment	5.91 (3.77)	4.68 (2.56)	1.23	3.516 (0.001)	5.49 (3.5)	4.58(2.7)	0.91	2.792 (0.006)

**Table 3:** Other acts of violence by intimate and non-intimate partners at baseline

Acts of Violence by IP or Non-IPs	Recent			Current		
	Usual Care	Intervention Group	p-value	Usual Care	Intervention Group	p-value
**Violence by IP**	**Yes (%)**	**Yes (%)**		**Yes (%)**	**Yes (%)**	
Refused to use a condom	104(73.2)	115(81.6)	0.092	105(73.9)	124(87.9)	0.003*
Tried to force me to get pregnant	35(24.6)	40(28.4)	0.470			
Neglected financially	101(71.1)	99(70.2)	0.868	101(71.1)	110(78.0)	0.184
Chased from home	83(58.5)	63(44.7)	0.020*	67(47.2)	54(38.3)	0.131
**Violence by Non-IPs**						
Sexual abuse	14(9.9)	20(14.2)	0.267	10(7.0)	19(13.5)	0.072
Physically abuse	22(15.5)	39(27.7)	0.013*	15(10.6)	30(21.3)	0.014*
**Respondent’s Physical violence against IP**	9(6.3)	9(6.4)	0.973	7(4.9)	3(2.12)	0.201

* Significant at 5% Level

**Antepartum depression scores at baseline**: The usual care and intervention groups did not differ significantly in the proportions with EPDS score ≥ 13, 72.78%, 95% CI (66.9-80.5) versus 65.27%, 95% CI (56.9-72.5) respectively. The groups were not different in terms of the mean EPDS scores at baseline, 15.58, SD = 3.74 in the usual care versus 14.07, SD = 4.27 in the intervention group.

**GBV and antepartum depression post-intervention**: **Table 4** summarizes the total IPV and subscale scores post-intervention. After adjusting for baseline scores, there was a significant difference between the psychosocial intervention and the usual care groups in the total IPV and physical violence scores, with small effect size (ES) of partial eta squared = 0.196 and 0.305, respectively. A strong relationship between the pre-intervention and post-intervention total IPV and physical violence scores was also established, partial eta squared = 0.824 and 0.724, respectively. The difference in the post-intervention subscale scores for severe combined violence, emotional violence and harassment was significant after adjustment for pre-intervention scores (p < 0.001) but the effect sizes were negligible. A strong relationship between the pre and post-intervention scores in all the subscales was established; severe combined violence (partial eta squared = 0.819), emotional violence (partial eta squared = 0.770) and harassment partial eta squared = 0.758). There was a slight decline in the proportion of those reporting other acts GBV by IPs and non-IPs in the intervention arm at the end of the study but these proportions were not significantly different in the two groups. EPDS scores declined in both groups between the baseline and the end of the study. The intervention group had significantly lower mean EPDS score compared to the usual care post-intervention, F (1,280) = 106.25, p < 0.001, with a medium between the groups ES of 0.500.

**Table 4:** IPV and antepartum depression scores post-intervention

CAS Measure	Intervention Group, n=141	Usual care, n=142	Between Groups Difference	95% CI of the Difference	F(df)	Partial Eta Squared
**Mean (SD)**						
Total IPV	17.70(11.12)	31.22(20.17)	13.51*	9.99-17.02	79.98(1,280)	0.196
Severe Combined violence	2.79(2.78)	4.51(4.24)	1.72*	0.95-2.49	15.78(1,280)	0.046
Physical violence	5.20(3.45)	6.76(5.8)	1.57*	1.52-2.60	144.2(1,280)	0.305
Emotional violence	8.52(5.56)	14.57(9.43)	6.09*	4.41-7.76	27.80(1,280)	0.078
Harassment	3.90(2.59)	5.55(3.24)	1.65*	1.02-2.28	30.81(1,280)	0.086
**EPDS Score**						
**Mean(SD)**						
Total EPDS	5.34(4.23)	12.46(4.22)	7.12*	6.21-8.03	106.25(1,280)	0.500

95% CI of the difference based on independent samples t-test

* Mean difference significant at 5% Level

## Discussion

To the best of our knowledge, this study is the first in Kenya to investigate the effectiveness of a psychosocial intervention for abused pregnant women in a primary healthcare setting. The interventionists were trained community health volunteers supported by the lead and co-investigators and two professional counsellors experienced in GBV. Human resource constraints particularly counsellors experienced in GBV in primary healthcare facilities in Kenya necessitated the utilization of non-professionals to deliver the intervention in this study. Some studies used community workers [44] social workers [34, 45] and mentor mothers [33, 35] to deliver interventions aimed at reducing IPV. This study thus, adds to the growing evidence that non-professional befriending models have their role in the spectrum of professional and non-professional responses to GBV [33]. The high response rate in our study is supported by findings that clients can disclose violence if healthcare providers play an active role [46], contrary to the perception that disclosure of violence an arduous task. It also points to the potential role that primary health care facilities can play in the identification and management of GBV during pregnancy [5]. Exposure to intimate and non- intimate partner violence before and during pregnancy is common hence, the need to accord GBV priority when addressing healthcare needs of pregnant women in Kisumu County. A recent study in the county found the integration of GBV services into ANC to be feasible and acceptable to both healthcare providers and users [3]. More than three-quarters of the participants in the intervention group completed three supportive sessions, which was relatively good for pregnant women in a low-income country where violence compounds their many competing needs and demands. The high completion rate in the intervention group may be suggestive of the intervention's acceptability to most of the participants. The intervention and usual care groups showed a reduction in scores for total IPV, severe combined violence, physical and emotional violence as well as harassment post-intervention but the reduction was much higher in the intervention group. The psychosocial intervention had a small but not negligible effect on total IPV (ES = 0.2) and physical violence (ES = 0.3). This finding indicates that the intervention had beneficial effects of lowering the mean score of the total and physical violence. Similar interventions reported reduction in IPV scores [35, 44], decline in the proportion of women reporting violence [47], reduction in minor physical violence [48] and declining trends in IPV [33, 34].

The effect of the intervention on severe combined violence, emotional violence and harassment, though significant, produced negligible effect sizes. The intervention spanned a period of 4 months and the last interview took place immediately after the last psychosocial session. Given that ending violence can be a long-term and complex process [49], it is possible that that the relatively short duration resulted in the failure to find meaningful effects for the three subscales. There was a reduction in the proportion of those reporting other forms of intimate and non-intimate partner violence in both groups post-intervention. Asking women about abuse and offering referrals increases awareness and motivates help-seeking behavior resulting to a reduction in violence [42, 50]. This might explain the reduction in the proportions reporting other forms of IPV and non-intimate partner violence in this study. The proportion reporting other forms of IPV and non-intimate partner violence, however, did not differ significantly between groups. We found no significant difference in the proportion of women reporting physical violence by a non-intimate partner in both groups post-intervention despite finding the intervention effective in lowering physical violence from an intimate partner. The safety planning protocol used in this study [43] appears to be more applicable in the context of violence in an intimate relationship, thus, the possibility that women in the intervention group experienced challenges in the applying of the safety-planning component beyond the intimate relationship set up. Future studies with abused pregnant women, therefore, need to find ways to factor in non-intimate partner violence into safety planning. Although the intervention group was encouraged to save money and consider acquiring valuables, this may take time given that part of abusive partners' strategy is to deprive their victims of finances in order to maintain a cycle of dependency. Thus, reduction in economic violence (perceived financial neglect) may require elaborate approaches that target economic empowerment. Approaches targeting norms, beliefs and other gender-related inequalities in the wider community known to contribute to physical and sexual violence by non-intimate partners are potential components in primary prevention, which can complement facility-based efforts.

The intervention group had significantly lower mean EPDS scores post-intervention compared to the usual care, with a substantial effect size on the reduction of antepartum depression (0.5). GBV is associated with many adverse effects including depression and poor quality of life and many abused women suffer in silence and self-blame. The psychosocial intervention offered women a rare opportunity to have someone validate their feelings, encourage and listen to them in an empathetic and non-judgemental environment. The cathartic effect of releasing pent-up tension may explain the favourable results [48]. The safety component of the intervention may have resulted to increased self-efficacy, which reduced the sense of helplessness, despair and antepartum depression. Further, knowing that GBV affects a substantial number of other pregnant women and the sense of increased support may have borne relief besides reducing the feeling of stigma and isolation associated with poor mental health. Previous studies that utilized advocacy and empowerment [34, 48] or mentorship [33,35] reported a reduction in depressive symptoms. The findings of this study must be viewed in the light of the constraints of this investigation. We could not be overly persistent in our attempts to negotiate appointments for participants who missed their ANC visits and by extension the psychosocial support session due to safety reasons. Majority of the participants in the intervention arm (77%) however completed all 3 supportive sessions, 16% completed 2 sessions and 7% completed 1 session before taking the last interview. Similar studies with pregnant women reported delivering one 30 minutes session of the intervention before the post-intervention interview [45, 48]. The attendance rate for the intervention in our study is commendable considering the percentage (58.7%) of pregnant women in the study area who manage to make at least four ANC visits during pregnancy [2]. This study investigated the effect of a psychosocial intervention compared to usual antenatal care, on GBV and antepartum depression irrespective of the number of psychosocial sessions the participants received. The study did not recruit women who arrived for ANC accompanied by their partners or other family members, which may be indicative of being in a controlling and abusive relationship. We, therefore, do not know how these women would have responded to the intervention.

## Conclusion

A facility based psychosocial intervention aimed at secondary prevention of GBV and improvement of mental health in pregnant women holds promise. There is potential for primary healthcare facilities in resource-constrained settings to can take advantage of local capacity in delivering focused non-specialized psychosocial support to pregnant women experiencing GBV.

### What is known about this topic

- Assessment for GBV is not part of routine antenatal care in Kenya;
- Specialized counseling services provided by trained professionals are rarely available to violated women in low and middle-income countries;
- Facility-based interventions carried out predominantly in developed countries with the aim of reducing GBV and improving the well-being of abused women hold promise.

### What this study adds

- A psychosocial intervention can help reduce the mean total and physical IPV as well as antepartum depressive symptoms during pregnancy;
- Trained community health volunteers can be used to provide focused non-specialized psychosocial support to abused pregnant women;
- GBV interventions targeting abused pregnant clients can be nested within the ANC clinics.

## Competing interests

The authors declare no competing interests.
